# Ketogenic Diet and Neuroinflammation: Implications for Neuroimmunometabolism and Therapeutic Approaches to Refractory Epilepsy

**DOI:** 10.3390/nu16233994

**Published:** 2024-11-22

**Authors:** Daniela Guerreiro, Anabela Almeida, Renata Ramalho

**Affiliations:** 1 Nutrition Lab, Egas Moniz Center for Interdisciplinary Research (CiiEM, U4585 FCT), Egas Moniz School of Health and Science, 2829-511 Caparica, Portugal; danielaguerreiro41@gmail.com; 2 Nutritional Immunology—Clinical and Experimental Lab (NICE Lab), Clinical Research Unit, Egas Moniz Center for Interdisciplinary Research (CiiEM, U4585 FCT), Egas Moniz School of Health and Science, 2829-511 Caparica, Portugal; 3 Serviço de Nutrição do Hospital Garcia de Orta (HGO), 2805-267 Almada, Portugal; aalmeida@ulsas.min-saude.pt

**Keywords:** immune cells, immunometabolism, ketogenic diet, ketone bodies, neuroinflammation, neuroimmunometabolism, refractory epilepsy

## Abstract

Refractory epilepsy, characterized by seizures that do not respond to standard antiseizure medications, remains a significant clinical challenge. The central role of the immune system on the occurrence of epileptic disorders has been long studied, but recent perspectives on immunometabolism and neuroinflammation are reshaping scientific knowledge. The ketogenic diet and its variants have been considered an important medical nutrition therapy for refractory epilepsy and may have a potential modulation effect on the immune system, specifically, on the metabolism of immune cells. In this comprehensive review, we gathered current evidence-based practice, ketogenic diet variants and interventional ongoing clinical trials addressing the role of the ketogenic diet in epilepsy. We also discussed in detail the ketogenic diet metabolism and its anticonvulsant mechanisms, and the potential role of this diet on neuroinflammation and neuroimmunometabolism, highlighting Th17/Treg homeostasis as one of the most interesting aspects of ketogenic diet immune modulation in refractory epilepsy, deserving consideration in future clinical trials.

## 1. Introduction

Epilepsy is one of the most common neurological diseases, with a worldwide prevalence of 0.5–1% and a lifetime incidence of 1–3% [1]. Despite the first line of treatment being the pharmacological approach, 30% of patients maintain convulsive crises. In these cases, it becomes necessary to resort to non-pharmacological approaches to control epileptic seizures [2]. There are several non-pharmacological approaches to control refractory epilepsy, such as vagus nerve stimulation, responsive neurostimulation, surgery, and the ketogenic diet (KD) [1].

The relationship between diet and epilepsy has been an ongoing research topic since 500BC. Hippocrates tested a hypothesized link between a complete fast and an individual with seizures, resulting in a positive outcome [3]. In 1920, Russel Wilder published two articles on the effects of ketonemia on epilepsy [4]. He first reported on the interest of fasting for patients with epilepsy, suggesting that the benefits of fasting on seizures may depend on ketonemia [4]. Afterwards, it was suggested that equally good results could be obtained with a diet very rich in fat and very low on carbohydrate, once it would provoke ketogenesis [4]. The effects were observed on three patients over time. Following Wilder, nine papers were published, involving more than 400 patients with epilepsy. The use of the ketogenic diet rapidly spread in the 1920s [4]. Despite some underlying mechanisms of the KD still need to be understood, KD therapies are now evidence-based treatments of refractory epilepsy. Under physiological conditions, glucose is the main energy substrate. However, during a fasting period, glucose levels decrease, and the energy substrate becomes fat, through the oxidation of fatty acids in the mitochondria. Consequently, Acetylcoenzyme A (Acetyl-CoA) is produced, which is converted into ketone bodies (KB) in the liver. In this situation, this is the alternative energy source [5,6]. The KD aims to mimic the effects of fasting on the body, as ketonemia has a therapeutical effect in several clinical conditions. According to current scientific evidence, a 2018 consensus panel recommended the implementation of the KD in children with refractory epilepsy, that is, after the unsuccessful treatment of seizures after the administration of two different antiepileptic drugs [7].

Nutrition has been considered an important modulator of the immune system, affecting immune cells’ lives in all dimensions [8]. The modulation of immune cells ‘metabolism by nutrients—immunometabolism—has critical implications for immune cells’ phenotype and biological functions, as demonstrated in cancer and infection [9]. Neuroinflammation has also been recognized as important for epilepsy pathophysiology; however, several questions remain to be answered [10]. Here, we present a comprehensive revision of immunometabolism and neuroinflammation in refractory epilepsy and gather evidence on the role of the KD in modulating inflammatory responses on this condition. We have also gathered a list of active clinical trials on the effects of the KD on epilepsy, which use immunological markers as endpoints.

## 2. Epilepsy

Epilepsy is a chronic brain disorder characterized by a systematic predisposal to develop seizures, which are short episodes of involuntary movement that can affect part or the whole body, sometimes accompanied by loss of consciousness and control of bladder or bowel function. As defined by the International League Against Epilepsy, the occurrence of two or more unprovoked seizures defines epilepsy and epilepsy resistant to pharmacological therapy is defined by refractory epilepsy [11]. Epilepsy is divided in two etiological categories: focal or generalized. Focal seizures originate within networks limited to one hemisphere. They are further subdivided, primarily based upon the clinical signs, symptoms and EEG location, into motor seizures, sensory seizures, autonomic seizures, focal seizures without impairment of awareness and focal impaired awareness seizures. Generalized seizures can be conceptualized as originating at some point within, and rapidly engaging, bilaterally distributed networks. Awareness may be impaired, and this impairment can be the initial manifestation [12].

In 50% of global cases, the etiology is not clarified, meaning that six in 10 patients are diagnosed with idiopathic epilepsy [11]. In childhood, the idiopathic form of epilepsy manifests itself without visible neurological signs, while the acquired form is related to identifiable structural lesions in the brain, resulting from trauma, tumors, infections, hippocampal sclerosis, cerebrovascular, immunological syndromes, perinatal or prenatal injuries and childhood disorders. On the other hand, cryptogenic epilepsy remains an etiological enigma [13]. Epileptic syndromes are characterized by pharmacological resistance, epileptic polymorphism and severe alterations in the electroencephalogram patterns, and its neuropsychiatric manifestations range from mild to severe [13]. These include neurological impairments, mental retardation, sensory and communication deficits and psychiatric, motor and behavioral aspects [13,14]. A complex combination of several factors, such as epigenetic modifications, brain injuries, environmental factors or pollutants [15] and diet composition [16], is involved in the occurrence of epilepsy. These factors may reconfigure brain circuits in a unique and individualized way, ultimately leading to epileptic disorders [13].

The other half of patients diagnosed with epilepsy are diagnosed with a known etiology, which may vary between brain tumors, stroke, brain infection, severe head injury, congenital abnormalities, brain damage occurred from perinatal or prenatal injuries and certain genetic syndromes [11]. The neuropathological mechanisms of epilepsy are only partly understood and may include general neurochemical and blood–brain barrier dysfunction, the generation of free radicals [17,18], the release of excitotoxins such as glutamate and alternations in energy metabolism [19]. Such neuropathology is believed to be the result of long-term alterations in the brain neuronal network [20].

Classic epilepsy treatment is based on pharmacological intervention [13]. However, other therapies include surgery and vagus nerve stimulation (VNS) [21]. Despite these therapies, approximately 30% of patients with epilepsy remain with uncontrolled seizures and become resistant to medication [13]. Under a circumstance of resistance, adding a new antiepileptic drug shows no beneficial effect on seizure control. Every intervention requires an individualized approach according to the patient, his medical history and the kind of epilepsy and seizures. Every antiepileptic drug must be carefully selected according to its pharmacokinetic proprieties, safety, and tolerability, as well as the specific patient’s comorbidities and circumstances [20]. VNS is usually an option for patients that do not meet the requirements for surgery [22]. In this approach, there is a transmission of electrical impulses from a gyrator to the vagus nerve, aiming to alter the functional connectivity of several regions of the brain by changing the synaptic plasticity, and promoting a change on the release of neurotransmitters like serotonin, gamma-aminobutyric acid (GABA) and norepinephrine, as well as promoting an anti-inflammatory effect [22]. The VNS objective is to reduce excitatory activity and reduce the occurrence of seizures in epileptic patients. Surgery is an option if the etiology of epilepsy is known and should be seen as a last line of therapy [23]. It removes or suppress the region of the brain responsible for the crises and its efficacy is dependent upon the type of epilepsy [24]. Based on clinical trials, most patients experience a decrease in the number and intensity of seizures [25]. The Internation League Against Epilepsy (ILAE) recognizes over 30 epilepsy syndromes, each defined by a distinctive combination of clinical features, signs and symptoms and electrographic patterns [12].

Although epilepsy research is ongoing, the mechanisms underlying this disease are not completely understood and a gold standard therapy for all patients is not available. Living with uncontrolled epilepsy has a negative impact on the quality of life of patients and their caregivers [26], so exploiting the pathophysiological mechanisms deeper and generating strong evidence for alternative therapeutic strategies is imperative.

## 3. The Role of Immune System in Epilepsy

### 3.1. Neuroinflammation and Epilepsy

The central role of the immune system on health and disease has been long accepted. Inflammation is a hallmark in cardiovascular disease, obesity, diabetes, cancer, aging and neurological disorders [27,28,29,30]. Even its role in diseases associated to climate change have raised interest from the scientific community [31]. The term “neuroinflammation” has been attributed to the inflammatory response—and its players, cytokines, chemokines, cells and reactive oxidative species—that occurs in the brain and the spinal cord. This has been extensively revised in [32,33]. Neuroinflammation involves the development of an immune response mediated by pro-inflammatory cytokines (IL-1β, IL-6 and TNFα), chemokines (CCL2, CCL5 and CXCL1); secondary messengers (NO and prostaglandins); reactive oxygen species (ROS), which are produced by microglia—innate immune cells responsible for the primary immune surveillance and macrophage-like activities of the central nervous System (CNS)—and astrocytes [32]. This is a complex concept, involving the players and endothelial cells, perivascular macrophages and the interaction between T cells and resident CNS cells [33]. The central point is that, as in all intricate networks of the immune system, there are positive and negative aspects of neuroinflammation, and the intensity of the response needs also to be considered [34]. Controlled and brief inflammatory responses are generally positive—beneficial to the host [32]. This type of response is generally of medium or low intensity, as well as being transient. These beneficial inflammatory responses are characterized by the production of IL-1 and IL-4, and culminate in neuroprotection, tissue repair, enhanced plasticity and the reorganization of host priorities—mostly important, learning with antigen encounters and immunological memory are achieved (e.g., after infection) [32,35]. On the other hand, high intensity, transient and chronic inflammatory responses are generally maladaptive—negative to the host [32]. Representatives of this type of neuroinflammation are traumatic brain injuries, aging, stress and neurodegenerative diseases [32]. These are marked by the production of IL-1, IL-6, TNF, CCL2, ROS, iNOS (inducible nitric oxide synthase) and INFγ, ending in neuronal damage, reduced plasticity, cognitive impairment, anxiety, depression and excessive collateral damage to normal tissues [10,32]. Aside from the production of chemical mediators, neuroinflammation is also characterized by the functional activation and proliferation of microglia and astrocytes, infiltration of monocytes-macrophages and T lymphocytes (including Treg) and neuronal cell death due to neurotoxicity [36].

Neuroinflammation has been recognized as important for epilepsy pathophysiology [10]; however, several questions remain to be answered. It is accepted that neuroinflammation is involved in neuronal hyperexcitability and epileptic seizures, and that a cascade of neuroinflammatory reactions may be triggered by prolonged epileptic seizures. Table 1 summarizes the effect of the main neuroinflammation players on epilepsy.

Data from both animal and human studies have contributed to the enlightenment of neuroinflammation’s role in seizures and epileptogenesis [10,33,37,38,39]. Pathogens, self-antigens or brain tissue injury may drive neuroinflammation through the activation of microglia and astrocytes, leading to the pro-inflammatory mediator’s release and the migration of peripheral immune cells (and serum albumin) from blood to the brain [37]. This subsequently induces an imbalance between glutamatergic signaling and GABAergic signaling and contributes to epilepsy [40,41]. Several signaling pathways are upregulated in epilepsy and involved in neuroinflammation and epileptogenesis. Table 2 gathers the main upregulated signaling pathways and their mechanisms in epilepsy. Most of these have been studied in mouse models of the disease.

Along with neuroinflammation, the role of immune cells, and especially subset phenotypes, in epilepsy is a hot topic [42]. In fact, metabolic alterations in immune cells change their phenotype, and ultimately, this affects biological function, leaving therapeutic space for the modulation of immune responses by nutrients. This is of such relevance for various cerebral diseases that the metabolism of immune cells—coined as immunometabolism—has recently evolved to neuroimmunometabolism, when considering its role in these pathologies [39].

### 3.2. Immunometabolism and Epilepsy

Immunometabolism refers to the mechanisms linking the immune system to metabolism [43,44]. Metabolic reprogramming of immune cells, mainly by the availability of some substrates/nutrients to sustain biochemical pathways that generate ATP for cell demands, conduce to phenotypic changes that alter the biological function of these cells [45]. This is why several chronic non-communicable diseases, where nutritional imbalances persist, present a more pro-inflammatory phenotype of immune cells [9,46]. It is well known that nutrients shape the metabolism of immune cells, as well as pathogens, environmental pollutants, aging and cancer. As for these conditions, in epilepsy, inflammation intersects metabolism, leading to the metabolic reprogramming of neurons and glial cells through their immunometabolic sensors [34,47]. Neuroimmunometabolism is, therefore, a modern concept that unravels this intersection of neuroinflammation and immunometabolism [39]. The peculiarity of the brain is that its demand for energy is clearly higher than that of other body organs, so metabolic control in this body area must be tightened. This is achieved by metabolic sensors (receptors, enzymes and transporters) that enable glial cells to expend energy in response to elevated neuronal demands and modulate glial inflammatory responses through metabolic and immune signaling pathway crosstalk [39]. Several immunometabolic sensors are implicated in this, as synthetized in Table 3.

**Figure 1:**
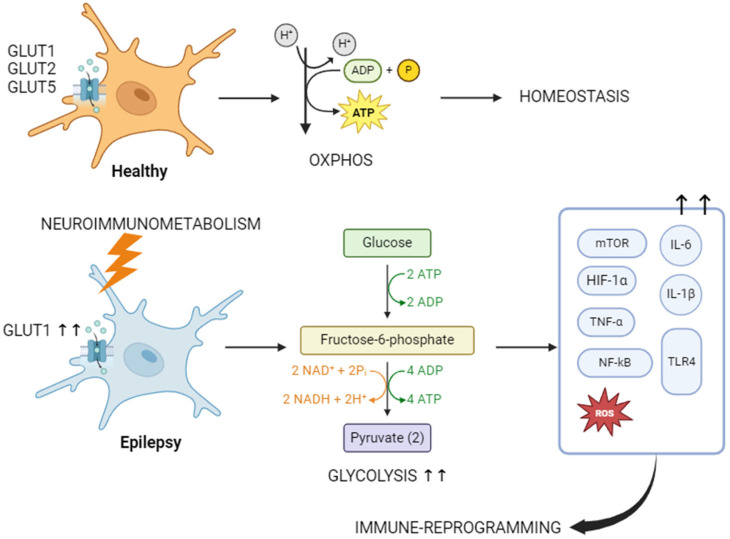
Immunometabolism and epilepsy. Impact of metabolic shift towards glycolysis on immune-reprogramming. HIF- hypoxia-inducible factor; IL—interleukin; mTOR—mammalian target of rapamycin; TLR—toll-like receptor; TNF—tumor necrosis factor. Black arrows mean increase production. Created in BioRender.com.

In a healthy brain, glucose metabolites are the main neuronal energy source. GLUT1, GLUT2 and GLUT5 are responsible for the transport of glucose from astrocytes, microglia and oligodendrocytes to neurons (entering via GLUT3) [48]. The main metabolic pathway in this situation is OXPHOS [10,34]. The depolarization of neurons is assumed by amino-acid metabolism, through conversion of glutamine into glutamate in the glutamate–glutamine cycle in astrocytes [49]. Structural support to neurons is provided by lipid metabolism. Neuroimmunometabolism disrupts the homeostasis of the healthy brain (Figure 1). This dysregulation is the result of a glucose metabolism shift towards aerobic glycolysis [50], increasing GLUT1 expression [51] as well as TNF, AMPK, mTOR, HIF1α and NF-kB [52]. Simultaneously, glutamate, tryptophane and arginine enter the amino-acid metabolism, increasing TNF, IL1β, ROS, mTOR, NADPH and NOX [53,54]. Lipid metabolism is also altered, with marked fatty acid toxicity due to NF-kB, PI3K/AkT, TLR-4 and ROS activation. All this reprogramming contributes to neuroinflammation and neuronal death. A recent review extensively explored this issue [39].

**Figure 2:**
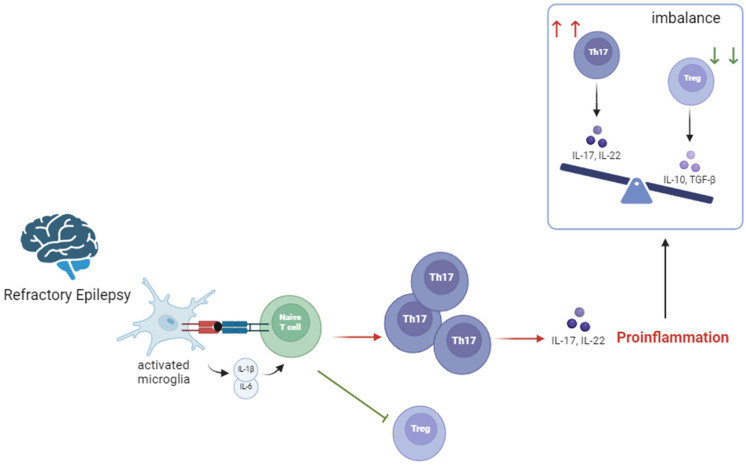
Th17/Treg imbalance in refractory epilepsy. IL—interleukin; TGF—transforming growth factor; Th—helper T lymphocyte. Red arrows mean increase in number and green arrows mean decrease in number of cells. Created in BioRender.com.

Immunometabolism in epilepsy is marked by all this reprogramming. Characteristic features of immune modulation in epilepsy include an activated and pro-inflammatory T lymphocyte response (both CD4+ and CD8+), interaction between T lymphocytes and activated microglia, an increase in IL-7 CD4+ and CD8+T-Lymphocytes, a reduction in LAG3+CD8+T-lymphocytes in peripheral blood, and increased IL-1B, IL-8, IL-12p70 and MIP-1β in epileptic focus [55]. Specifically, when naïve T lymphocytes encounter activated secreting IL-6 and IL-1β microglia, conversion into Th17-Lymphocytes occur, while conversion into regulatory T lymphocytes (Tregs) is inhibited. The balance between Th17 and Tregs is crucial. While Tregs in the brain (CD4+Foxp3+ T lymphocytes) regulate immune cell homeostasis by suppressing the immune response, promoting immune tolerance and inhibiting unnecessary inflammation, Th17 are essentially proinflammatory through IL-17 secretion and involved in autoimmunity and inflammatory responses [56]. Whereas the healthy brain exhibits a predominance of Tregs, in the epileptic brain, Th17 responses are dominant (Figure 2). So, Tregs are inversely correlated with disease severity [57].

Nutrients may modulate immune responses in health and disease, contributing to important phenotype changes. The role of nutrients in modulating neuroimmunometabolism remains an important field of research in brain diseases. Below, we review the biochemical and nutritional aspects of the ketogenic diet (KD) and gather data on the clinical relevance of this therapeutic diet for immunometabolism and neuroinflammation.

## 4. Ketogenic Diet: Modulating Inflammatory Responses in Epilepsy

The KD is a diet-based therapy for epilepsy, especially for children with drug-resistant disease. The KD has a very high fat and low carbohydrate profile—fat represents around 90% of the total energy intake (TEI) in this diet, and protein around 6% of TEI [58]. This severe glucose restriction triggers a systemic shift from glucose metabolism toward the metabolism of fatty acids using ketone bodies, such as acetoacetate and β-hydroxybutyrate, as a main substrate for energy [58]. All variants of the KD activate shifts in body energy metabolism towards an increased use of dietary fat and adipose stores for energy generation [59].

### 4.1. KD Variants

There are four main forms of the ketogenic diet—The classic ketogenic diet (CKD), the medium-chain triglyceride ketogenic diet (MCTD), the modified Atkins diet (MAD) and low glycemic index treatment (LGIT). The CKD is the most restrictive and inflexible dietary pattern. All the other KD variants aim for the same result but have small composition differences compared to the CKD that improve their adherence, palatability, enjoyability and diversity [58,59,60]. Extensive comparison between these four variants can be found in the literature [58,61,62].

The CKD was first introduced in 1921 by Russel Wilder [63]. The main fat source in this KD variant is long-chain triglycerides—usually in a 4:1 ratio (for every four portions of fat, one portion of protein and carbohydrates must be added) [64]. As a general guideline, 80–90% lipids, 6–8% protein and 2–4% carbohydrates is used to induce ketosis. In children, whose protein needs are higher compared to adulthood, the ratio can be adapted to 3:1 or 3,5:1 [2,59,65]. The daily intake of multivitamin complexes and mineral supplements is strongly recommended, due to an unbalanced eating pattern and lack of fruits, vegetables, milk and dairy products [1]. The KD represents a highly restrictive diet which, normally, is started with the patient hospitalized [60,66]. The CKD should be initiated after a fasting period of 12–48 h. Then, protein restriction to 1 g/kg of body weight, fluids restriction to 60–75 mL/kg of body weight and reduced dietary calorie level to 80–90% of the estimated daily requirements is implemented [59]. The restrictive profile of the CKD is considered one of the main reasons for low adherence. In a series of 150 children and adolescents from the Johns Hopkins KD center, 17% of patients discontinued the CKD in the first 3 months of treatment, and 45% discontinued the treatment in the first year, due to its restrictiveness or insufficient clinical effects (improvement considered below expectations) [59]. To increase adherence, some KD centers initiate the CKD without previous fasting and hospitalization using a “gradual ratio slow initiation method” (gradually increase of fat). This alternative allows initiation without less restrictions in the hopes of reducing the adverse effects, increasing adherence to CKD [59,67]. In patients fed via gastrostomy tubes or in those in intensive care unit settings, the CKD is available via commercial formula preparation, and it can be considered as an option [59]. It was reported that in infants fed with the CKD via liquid formula, the compliance was superior, compared to solid-fed patients [59,66].

The MCTD is a variant first introduced in 1976 by Huttenlocher et al. [5] and modified in 2008 by Neal et al. [68]. This is a more flexible KD variant, which defends that medium-chain triglyceride lipids are responsible for producing more ketones per gram compared to long-chain triglyceride lipids [69]. The objective of the MCTD is to allow a high ketogenic potential from the lipids consumed—thus, it is possible to reduce fat intake and increase the amount of protein and carbohydrates, achieving the same state of ketosis [69,70]. With a lipid content of 30–60%, 10% protein and 15–19% carbohydrates, it allows better palatability, the higher supply of micronutrients and better management of blood lipid profile—e.g., cholesterol and LDL [67,70]. Clinical efficacy is maintained, and the main difference is the preference for medium-chain triglycerides—especially octanoic (C8) and decanoic (C10) fatty acids—in opposition to long-chain triglycerides. This variant promotes more pronounced gastrointestinal effects, resulting in less acceptance and implementation by health professionals [59,67].

The modified Atkins diet (MAD) is a variant that represents a less restrictive and more palatable adaptation, showing a 1:1 ratio. It only limits carbohydrate intake (10–20 g/day in children and 15–20 g/day in adults) and does not limit the daily intake of other macronutrients. Since it allows for more flexibility, it can be started at home, without hospitalization [67]. A distribution of macronutrients based on 65% lipids, 25% protein and 10% carbohydrates is recommended on this variant [64,71]. As with the other KDs, the daily intake of multivitamin complexes and calcium carbonate supplements is strongly recommended [1]. The MAD diet has higher tolerability and flexibility. However, it is generally reported to not reach the same increase in ketonemia levels as other variants [59,67].

The low glycemic index treatment (LGIT) is the most recent and flexible variant to date and allows for a daily an ingestion of 40–60 g of carbohydrates from foods with a glycemic index lower than 50. Lipids represent 60% of the total intake and proteins are found in a higher quantity compared to the other variants, representing 20–30%. Carbohydrates are quantified to a value of 10% and it is shown to represent a 1:6 ratio. There are no limitations on the introduction of liquids or calories, and as long as foods with a glycemic index lower than 50 are included, like meat, dairy products, some fruits, whole grains, nuts, and legumes like beans and chickpeas. Compared to the other variants, such as the CKD, the low glycemic index diet does not allow the production of such a high amount of ketone bodies, but on the other hand, it provides better tolerance and adherence and lower gastrointestinal side effects [1].

Current evidence suggests that only strict long-term adherence to the KD may lead to a fundamental shift from a glucose-based metabolism to a nutritional ketosis state [72]. The duration and the adherence to the protocol is mandatory to observe clinical efficacy for drug resistant epilepsy. To maintain this state of ketosis, any KD must be restricted in carbohydrates and enriched in fat, and it should provide adequate protein to ensure growth and protect lean body mass. The decision to use the KD in refractory epilepsy depends on the availability of the patient and their caregivers and medical condition. Unlike pharmacological treatment, the KD represents a multidisciplinary therapy that requires knowledge, support and cooperation from everyone involved—including from caregivers and healthcare professionals [59].

### 4.2. KD: Metabolism and Anticonvulsant Mechanisms

At the beginning of KD therapy, the blood glucose concentrations drop and stabilize, preventing postprandial insulin release. The body enters a catabolic state where glycogen stores are depleted. The body is forced into endogenous glucose production, especially in the liver (gluconeogenesis), using the amino acids alanine, glutamine, lactic acid and glycerol. When gluconeogenesis is not sufficient to meet the metabolic demands, free fatty acids are mobilized from fat tissues to be used as a primary source of energy. As glucose is not readily available, the brain is able to use ketone bodies generated by the oxidation of free fatty acids in the liver. Despite having a minimum requirement for glucose, when ketone bodies’ concentration in the blood reaches the range of 2–4 mM, 60% of the brain’s energy requirements are met [59]. During ketosis, the energy produced by the oxidation of fatty acids in mitochondria results in a production of large amounts of acetyl-CoA. The accumulation of acetyl-CoA leads to the synthesis of acetoacetate and β-hydroxybutyrate. After entering the brain, ketone bodies are metabolically converted into acetyl-CoA before entering the tricarboxylic acid cycle in the mitochondria of the brain, which ultimately leads to the synthesis of ATP [20]. The stable nutritional ketosis provides a steady fuel source for the energy-intensive tissues such as brain and muscle, preventing the likelihood of a disruption in energy availability [59].

Despite the anticonvulsant mechanisms of the ketogenic diet not being completely understood, it is theorized that ketone bodies and polyunsaturated fatty acids are key mediators in brain energy metabolism, oxidative stress, ion channel variations and neurotransmitter variations. Presumably, these mechanisms influence neuronal activity and play a major role in the anticonvulsant effects of the KD [20]. According to the current literature, brain tissue under the influence of a ketogenic diet becomes more resistant to metabolic stress. There is a decrease in glucose consumption and a higher production of glycolytic ATP, which induces potassium channels sensitive to ATP opening. Consequently, there is a hyperpolarization of the neuronal membrane, leading to a decrease in electrical excitability in the brain. This prevents the excessive firing of neurons and regulates the seizure threshold in the brain. In addition, the increase of ketone bodies and fatty acids may regulate neuronal membrane excitability, activating two pore domain potassium channels, which shows to be another anticonvulsant mechanism of action of the ketogenic diet [20]. There are several neurotransmitters and neuronal pathways that are currently studied to determine the underlying anticonvulsant mechanisms.

GABA is the main inhibitory neurotransmitter in the central nervous system and plays a key role in the genesis and spread of seizure activity [73]. It is considered that the ketogenic diet leads to the activation of the glutamic acid decarboxylase, which induces the synthesis of GABA—a neurotransmitter responsible for reducing the neuronal excitability in the brain. In parallel, KD shows evidence to alter GABA’s transaminase activity, leading to a decrease in the degradation of GABA. Glutamate is an essential neurotransmitter and represents the most abundant amino acid in the mammalian brain. This neurotransmitter plays an essential role in several processes, such as learning, memory, cognition and emotion [13]. It is released by glutamatergic neurons into the extracellular space and acts on ionotropic and metabotropic receptors. An imbalance between excessive glutamate and/or inadequate GABA can result in overexcitation of the central nervous system, predisposing the occurrence of seizures, and glutamatergic dysregulation leads to the accumulation of glutamate in the synapse, as well as the overactivation of glutamate receptors, resulting in excitotoxicity and, eventually, cell death. High levels of glutamate in the brain appear to make the brain more susceptible to seizures, associating glutamate with the development of epilepsy. However, the result of studies on the effect of the KD on glutamate levels are inconclusive—some studies reveal that the ketogenic diet has an influence on glutamate levels, while other studies show no effect [13,20].

Agmatine is a neuromodulator that regulates multiple neurotransmitters and signaling pathways. Several studies have focused on elucidating the mechanisms underlying the neuroprotective effects of this molecule, which seems to be mediated by a reduction in oxidative damage [74,75,76]. It has been shown in mouse studies that the ketogenic diet increases the level of agmatine in the hippocampus. Agmatine is found in synapses and is an inhibitory neurotransmitter that exerts an anti-seizure effect by inhibiting various brain stimulating receptors, including N-methyl-D-aspartate, histamine and adrenaline receptors [75,76]. Higher levels of agmatine in the brain are associated with neuroprotective and anticonvulsant properties [75,76]. Furthermore, it has seen in mice that agmatine enhances the anticonvulsant effects of valproate and phenobarbital in mice subjected to seizures, without involving any pharmacokinetic interactions [77]. In another clinical study where seizures were induced by pentylenetetrazol in mice, it was documented that agmatine reduces the protective effect of vigabatrin, without influencing the pharmacological effect of other antiepileptic drugs. It is suggested that this neurotransmitter potentiates a beneficial effect when combined with other anti-epileptic drugs [20]. However, the mechanisms behind this effect of KD on agmatine remain unclear.

Monoamine neurotransmitters such as serotonin, dopamine and noradrenaline are crucial in modulating neuronal excitability and seizure activity. The interplay between these neurotransmitters and their receptors can significantly influence the anticonvulsant mechanisms of the KD. Current evidence supports that serotonin and dopamine receptors are present in many neural networks that are involved in seizures, and they are directly correlated with the excitability of neurons [20]. The KD has been reported to influence the levels of monoamine transmitters, resulting in an increase in brain serotonin levels and altered dopamine metabolism. In parallel, it is observed that it also affects the expression and sensitivity of serotonin and dopamine receptors, further modulating seizure thresholds. In combination, there is evidence that the ketogenic diet reduces neuroinflammation, helps to stabilize neuronal excitability, reducing seizure occurrences [20].

Polyunsaturated fatty acids provided by the KD play a significant role in activating peroxisome proliferator-activated receptors. Their activation has several beneficial effects like anti-inflammatory and antioxidant properties (key to reduce inflammation and oxidative stress); additionally, they play an important role in increasing energy reserves (their activation enhances the oxidation of fatty acids, leading to more efficient energy production, which is beneficial in a ketogenic diet once it relies on fats as a primary energy source) and act as a stabilizer of synaptic functions (by modulating the expression of genes, the neuronal hyperexcitability is reduced, which can help in controlling seizures) [20]. In summary, the KD, through the activation of peroxisome proliferator-activated receptors by polyunsaturated fatty acids, provides multiple neuroprotective and metabolic benefits, contributing to better energy management, reduced inflammation, enhanced synaptic stability and decreased neuronal hyperexcitability [78].

It has been observed that the KD can also upregulate calbindin, which has neuroprotective potential through its ability to buffer intracellular calcium. Other neuroprotective properties include the inhibition of apoptotic factors such as caspase 3 and the inhibition of transient pores in mitochondria. Overall, an increased energy production in the brain, an improvement of the expression of energy metabolism genes, an improvement of mitochondrial biogenesis and density and an increase of energy reserves in the form of phosphocreatine are noted [20]. It should be noted that KD efficacy is not limited to seizure control, but also affects neurobehavioral development, cognitive functions and sleep quality [59]. The neurobehavioral improvements comprise adaptability, gross motor movements, language and social interactions. The cognitive benefits comprise alertness, fine motor movements, language and social interactions, attention and global cognition. There are indications that KD improves sleep quality by increasing REM sleep, ultimately leading to an improved quality of life [59].

### 4.3. Impact of KD on Th17/Treg Homeostasis Disruption

Besides the clinical interest in the KD’s role in epilepsy—which, without doubt, deserves more clarification—the role of this restrictive diet on the immune modulation of inflammation and metabolism in this condition is of utmost importance. Considering the key features of the immune system on epilepsy (mentioned earlier), the Th17/Treg homeostasis is one of the most interesting regarding therapeutic approaches. IL-17 is mainly derived from Th17 cells and is the main effector cytokine of Th17 cells, but the astrocytes and microglia of the CNS also express IL-17 [56,57]. All Th17 cells carry the C-C chemokine receptor 6 (CCR6) and express the retinoid-related orphan receptor gamma t (RORγt), which is considered a key transcription factor for T h17 cell differentiation. Regulatory T (Treg) cells play the opposite role to Th17 cells in the immune response; they mediate immune tolerance and act as the key to suppressing the excessive inflammatory activity of Th17 cells (19). Treg cell differentiation is driven by the transcription factor forkhead box P3 (FoxP3), which is reduced in individuals with inflammatory neurological diseases, and reduced Treg cell function is associated with the upregulation of RORγt. IL-17 has been revealed to have a strong association with neuroinflammation. Data on the therapeutic role of balancing Th17/Treg came from animal and human studies developed not only for epilepsy [79,80,81,82] but for other neurologic diseases such as Alzheimer’s disease, autism spectrum disorder and depression [38].

Regarding the effect of the KD on Th17 and Treg, reduced levels of intestinal proinflammatory Th17 cells were found with KD-associated gut microbiota transplantations into germ-free mice [62], and decreased levels of T-bet, IFN-γ, RORγt and IL-17 and increased GATA3, IL-4, Foxp3 and IL-10 were found in the spinal cord and spleen of mice treated with the KD [83]. Although very interesting, those animal studies address the role of the KD and IL-17 on the gut microbiome [62] and autoimmune encephalitis [83]. Data regarding epilepsy are far scarcer.

**Figure 3:**
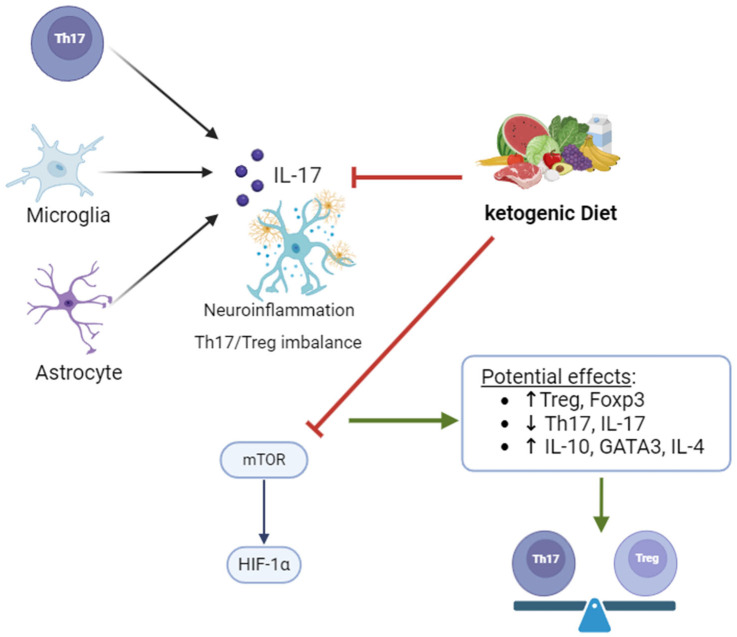
Role of KD diet in balancing Th1//Treg and reducing neuroinflammation. KD may potentially blockade mTOR/HIF-1a pathway, leading to Treg increasing and IL-10, IL-4 and GATA3 induction in refractory epilepsy. These mechanisms require further investigation. Foxp3—forkhead box P3; HIF—hypoxia-inducible factor; IL—interleukin; mTOR—mammalian target of rapamycin; Treg—regulatory T lymphocyte; Created in BioRender.com.

To the best of our knowledge, to date, only one study addresses this question in human studies. Ni et al. (2016) evaluated the effect of the KD on circulating levels of Th17/Treg cells, the plasma concentration of IL-17 and the mRNA levels of mTOR, HIF1α and Th17/Treg-associated factors in purified CD4+CD25+T and CD4+CD25 T cells [84]. In this study, 28 children (7 months to 12 years) were treated with an initial fasting stage of about 24–48 h, followed by a KD consisting of a lipid-to-non-lipid ratio of 4:1 (60–80 kcal/kg per day and 1–1.5 g/kg of protein, with potassium citrate, multivitamins and essential minerals supplementation). The findings showed that circulating Th17 T lymphocytes were increased in patients compared to healthy volunteers, as were the levels of IL-17A and RORγτ expression in CD4+CD25^−^ T lymphocytes. The downregulation of Treg was observed in patients. This was also observed in other studies [85,86]. Interestingly, in the study from Ni et al., this imbalance was reverted by the KD (IL 17A—26.89 ± 6.58 vs. 19.13± 5.94, *p* < 0.05; RORγτ—21.74 ± 5.97 × 10^4^ vs. 12.5 ± 4.5 × 10^4^, *p* < 0.001; Foxp3—12.50 ± 5.24 × 10^44^ vs. 22.60 ± 7.92 × 10^4^, *p* < 0.05; Treg—1.74 0.54% vs. 3.12 0.94%, *p* < 0.05) [84], probably through the inhibition of the mTOR/HIF-1α signaling pathway. These interesting results highlight the importance of designing clinical trials considering not only the effect of the KD on clinical outcomes, but also on the perspective of neuroimmunometabolism, and particularly, on the Th17/Treg balance, exploring the underlying mechanisms more deeply. Figure 3 highlights the potential impact of the KD on Th17/Treg homeostasis disruption in epilepsy.

We gathered information on ongoing clinical trials through clinicaltrials.gov (updated to July 2024) regarding the effect of the KD on epilepsy (Table 4). The only study intended to analyze the effect of the KD on immune-related parameters is NCT06310954. This study aims to analyze changes in inflammatory markers by KD treatment. Although no reference is made to cell counts, inflammatory parameters, at least, will be addressed by this study. The remaining ongoing studies give preference to the clinical aspects of disease control.

These data reinforce the need for in deep insight into neuroimmunometabolism when designing clinical trials on the effect of KD in epilepsy.

## 5. Potential Adverse Effects of the KD to Be Considered

Despite the type of KD, predictable and preventable adverse effects may occur in the short term [87]. Common adverse effects include gastrointestinal symptoms (up to 50%—diarrhea, constipation, nausea, vomiting and the exacerbation of gastroesophageal reflux), abnormal lipid parameters (14–59%), hypoglycemia (25%), growth failure, low levels of carnitine, bone disease, nephrolithiasis, selenium deficiency and neutropenia [67]. Less common or rare adverse (<0.5%) effects include cardiac abnormalities, pancreatitis and vascular changes [67]. Usually, gastrointestinal symptoms are managed symptomatically with proton pump inhibitors, laxatives and other symptomatic treatment as needed [58]. Most of these adverse effects will resolve after KD discontinuation. No increased risk of late reported cardiovascular disease, bone fractures or kidney stones have been found (except for those in higher risk of kidney stones) [88]. As lipid parameters are concerned, only a minority of children on KD will require medication, as deviations will improve spontaneously [89,90]. One or more episodes of hypoglycemia occur in approximately 25 percent of patients during the initiation week, mainly if there was a fasting period pre-diet. The inadequate supply of glucose to the brain and an inadequate control of hypoglycemia leads to headache, blurred or double vision, confusion, difficulty speaking, agitation, and possibly even coma [91]. Hyperuricemia, hypoproteinemia, hypomagnesemia, hyponatremia, hepatitis and metabolic acidosis are sometimes observed and should be monitored [67]. Most children on the KD fall into the lower weight and lower growth percentile. This growth failure appears to be more significant for younger children that follow the KD for a longer period. Height velocity is mostly affected when there is a higher level of ketosis [92,93]. Carnitine [67], selenium [94] and potassium [95] deficiencies should also be considered when the KD is administered. According to the International Ketogenic Diet Study Group clinical monitoring of these adverse events should be employed prior to KD initiation and during KD treatment. Prior to KD initiation, the following laboratory assessment is indicated: complete blood count with platelets, electrolytes (including bicarbonate, total protein and calcium), liver and kidney tests (including albumin, blood urea nitrogen and creatinine), fasting lipid profile, serum acylcarnitine profile, vitamin D level, urinalysis, urine organic acids (if diagnosis unclear) and amino acids (if diagnosis unclear) [67,96]. During KD treatment, free and total carnitine, selenium and, optionally, beta-hydroxybutyrate (BOH), urine calcium, creatinine, zinc and copper levels should be also included on the list [67,96].

## 6. Conclusions

The KD represents a promising therapeutic possibility for managing refractory epilepsy through its modulatory effects on neuroinflammation and neuroimmunometabolism. There is a clear link between these core concepts, leading to metabolic changes and the modulation of neuroinflammatory pathways. Th17/Treg homeostasis seems to be the most promising when considering immune modulation by KD in refractory epilepsy. Gathered ongoing research on the KD and refractory epilepsy revealed a lack of attention to immunometabolism and neuroinflammation, therefore highlighting the need of future clinical trials focusing on the effects of the KD on different aspects of the immune response, especially in Th17/Treg homeostasis. Accessing and understanding each patient’s metabolic profile may be a valuable tool in determining the cause of seizures and the optimal medical nutrition therapy. This could help clinicians offer focused and personalized nutrition care.

**Figure 4:**
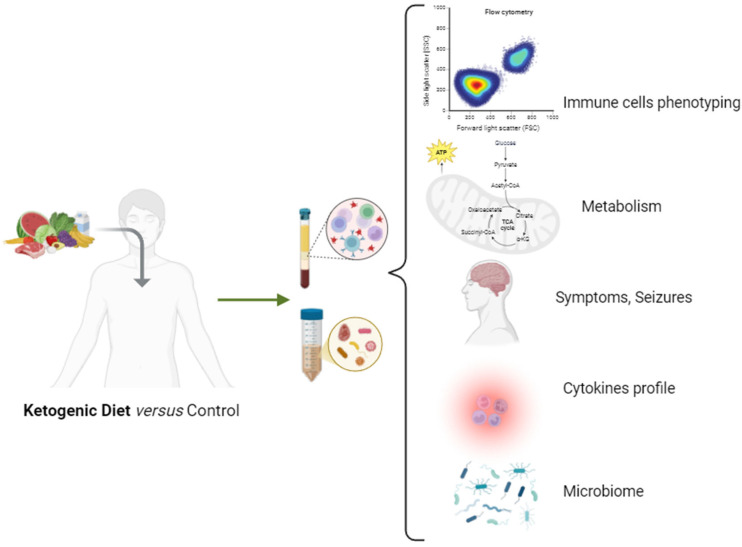
Future perspectives for clinical trials addressing the role of KD on refractory epilepsy. Created in BioRender.com.

The capacity to develop an individualized strategy may result in more effective and less restrictive nutritional therapy, reducing undesirable effects (Figure 4). 

## Figures and Tables

**Table 1:** Immune components secreted by microglia, astrocytes and neurons linking neuroinflammation to epilepsy.

Mediator	Secreted by	Mechanism
IL-1β	Microglia, Astrocytes, Neurons	Induce glutamate release Decrease glutamate reuptake Decrease GABA_A_ flows
IL-6	Microglia, Astrocytes	Induce glutamate release
IL-17	Microglia, Astrocytes, T lymphocytes	Promote infiltration of peripheral immune cells Inhibit GABA-induced inhibitory synaptic transmission
TNF-α	Microglia, Astrocytes	Promote infiltration of peripheral T-Lymphocytes Induce glutamate release Induce GABA receptor endocytosis
TGF-β	Astrocytes	Mediate albumin uptake Downregulate Kir4.1 channel Impair AQP4
HMGB1	Microglia, Astrocytes, Neurons	Interact with TLR4, increasing Ca^2+^ influx and activating the NMDAR by phosphorylating NR2B subunit Promote pro-inflammatory cytokines release
CCL2, CCL3, CCL4, CX3CL1, CXCL13	Microglia Astrocytes, Endothelial cells	Induce microglia activation Induce monocyte infiltration Promote neuron death through STAT3 and IL-1β signals

AQP—aquaporin; CCL—chemokine (C-C motif) ligand; CX3CCL1—fractalkine; CXCL—chemokine (C-X-C motif) ligand; GABA—gamma-aminobutyric acid; HMGB—high mobility group protein; IL—interleukin; Kir—killer-cell immunoglobulin-like receptor; NMDAR—N-methyl-D-aspartate receptor; NR2B—N-methyl-D-aspartate receptor subunit 2B; STAT—signal transducer and activator of transcription; TLR—toll-like receptor; TNF—tumor necrosis factor.

**Table 2:** Upregulated signaling pathways in epilepsy, involved in neuroinflammation.

Signal	Mechanism
TLRs	Induction of innate and adaptive immune responses, followed by neuronal hyperexcitability and epileptogenesis Activation of microglia Induction TNF-α and IFN-β
NLRP3	Induction of caspase-1 proteolysis Secretion of pro-inflammatory cytokines Epileptic neuron loss Seizures progression Induction of IL-1β and IL-18
COX-2/mPGES-1	Promotion of glutamate releasing by astrocytes, resulting in excitotoxicity Induction of PGE2 secretion by astrocytes and microglia
mTOR	Generation of monocytes and macrophages in marrow cavity Conversion of monocytes into macrophages by downregulation of CD115 expression Activation of T lymphocytes Activation of microglia BBB disruption Infiltration of peripheral immune cells into CNS Regulation of Th17 cells differentiation Mediation of IL-1β, IL-17 and TNF-α expression

BBB—blood–brain barrier; CD—cluster of differentiation; CNS—central nervous system; COX—cyclooxygenase; IL—interleukin; INF—interferon; mPGES-1; microsomal prostaglandin E synthase-1; mTOR—mammalian target of rapamycin; PGE2—prostaglandin E2; Th—helper T lymphocyte; TLR—toll-like receptor; NLRP3—NOD-, LRR- and pyrin domain-containing protein 3; TNF—tumor necrosis factor.

**Table 3:** Immunometabolic sensors involved in neuroimmunometabolism.

Type of Sensors	Examples and Expression
Sugar sensors	*Glucose* GLUT 1 brain vasculature oligodendrocytes microglia astrocytes GLUT3 neurons GLUT5 oligodendrocytes microglia astrocytes
Lipid sensors	*Cholesterol*, *aminophospholipids*, *gangliosides*, *sphingolipids* CD36, LDLR, VLDR, LRP1, TREM2 Microglia
Amino-acid sensors	*Tryptophan* Quinolinic acid, IDO and kynurenic acid microglia astrocytes *Glutamate* EAAT1, EAAT2 and astrocytic glutaminase microglia astrocytes *Arginine* Nitric oxid Microglia

CD—cluster of differentiation; EAAT—excitatory amino acid transporter; GLUT—glucose transporter; IDO—indoleamine 2,3-dioxygenase; LDLR—low-density lipoprotein receptor; LRP1—low-density lipoprotein receptor-related protein 1; TREM2—triggering receptor expressed on myeloid cells 2; VLDR—very-low-density lipoprotein receptor.

**Table 4:** Interventional ongoing studies addressing the role of KD on epilepsy.

Study	Phase	Participants	Intervention	Outcomes	Status	Completion Date
NCT04063007, EpiMICRO	NA	2 to 17 y, n = 60	KD (Single Group Assignment)	-Gut microbiota -Changes in the -DNA methylation in WBC -QoL -AE	Recruiting	2022—overdue, not completed
NCT05958160, TOPAMAD	Phase 2 Phase 3	9 mo to 3 y, n = 70	MAD vs. *Topiramate*	-Reduction in clinical spasms	Recruiting	2024
NCT02216500	NA	up to 50 y, n = 400	KD (Single Group Assignment)	-Epilepsy control response rate	Recruiting	2031
NCT06310954	NA	6 mo to 12 y, n = 59	KD vs. standard diet without any ketogenic restrictions	-Epilepsy control response rate -**Serum levels of inflammatory markers** -**Relationship between KD and inflammation**	Recruiting	2024
NCT05152771	NA	2 to 15 y, n = 26	KD (Single Group Assignment)	-Epilepsy control response rate -Cognitive changes -Behavioral changes -Motor developmental changes	Not yet recruiting	2025
NCT04274179	Phase 3	3 to 12 y, n = 40	MAD vs. standard diet without any ketogenic restrictions	-Epilepsy control response rate -Tolerability	Recruiting	2025
NCT06369571	Phase 1 Phase 2	>18 y, n = 22	MAD vs. replacement of 10% of saturated fat intake with polyunsaturated fat	-LDL changes -Epilepsy control response rate -AE	Not yet recruiting	2027

AE—adverse events; KD—ketogenic diet; LDL—low-density lipoprotein; MAD—modified Atkins diet; mo—months; NA—not available; QoL—quality of life; WBC—white blood cells; y—years. Bold text shows outcomes related to immune function.
